# Radial Distribution Study of Vitreous Barium Borate^1^

**DOI:** 10.6028/jres.064A.023

**Published:** 1960-06-01

**Authors:** Arthur Bienenstock, Aaron S. Posner, Stanley Block

## Abstract

X-ray diffraction, radial distribution studies of a 20 percent barium oxide, 80 percent B_2_O_3_ glass have been performed using both the atomic and electronic distribution functions. From these distributions, the average barium-barium distance has been determined as 6.76 A. This distance is in good agreement with that predicted by Levin and Block on the basis of a structural interpretation of immiscibility data.

## 1. Introduction

**Figure 1:**
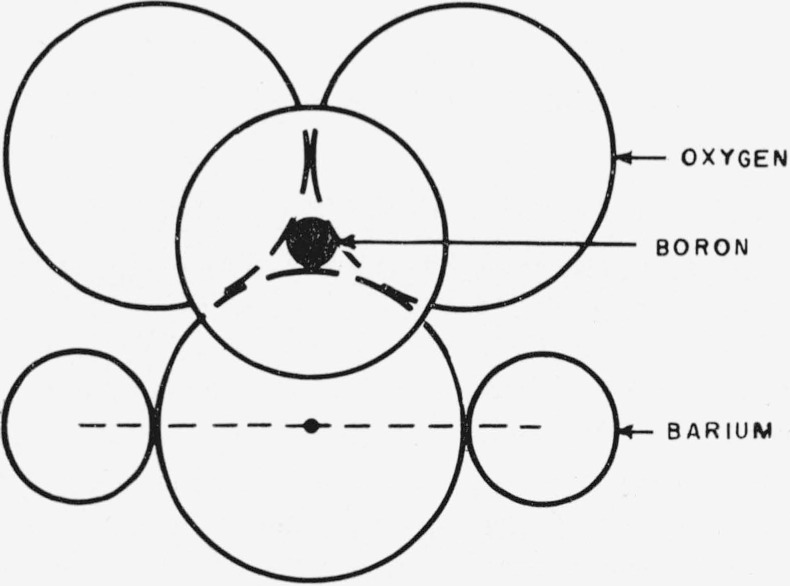
Type A coordination. Boron can be either in tetrahedral or triangular coordination. The Ba-Ba distance is the sum of the Ba–O distances.

Levin and Block [1] have attempted to explain immiscibility of alkali and alkali-earth metal oxides in borate and silicate glasses by means of crystal chemical principles. They considered two short-range structures proposed by Warren and Pincus [2] for the system at the limit of miscibility. For the particular system investigated here, BaO–B_2_O_3_, the type A structure, shown in figure 1, has the boron coordinated either triangularly or tetrahedrally by oxygen. Two bariums are bonded to the same oxygen with approximately a 180° bond angle. Using ionic radii, they calculate a separation of barium atoms of 5.50 A.

**Figure 2:**
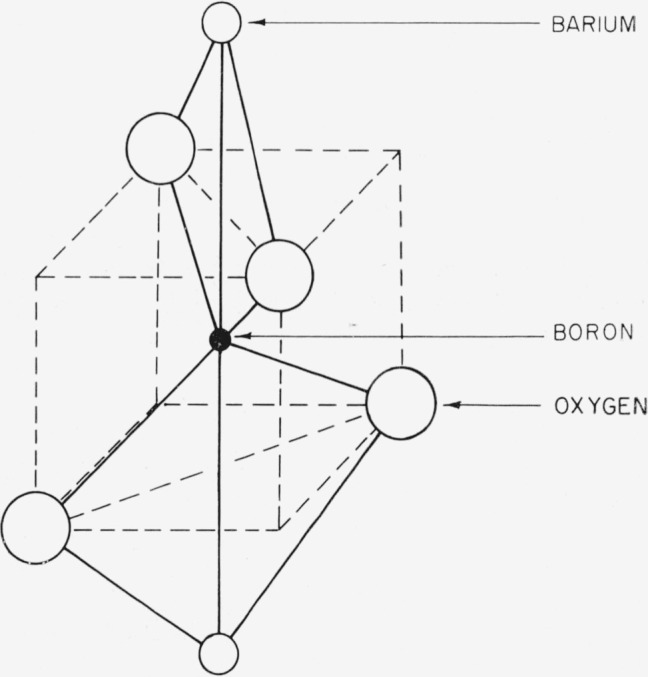
Type B coordination. Boron must be in tetrahedral configuration. The boron and barium polyhedra share edges.

The type B structure is shown in figure 2. Here, each boron is tetrahedrally coordinated by oxygen. The boron tetrahedron shares edges with a distorted barium cube. The predicted barium–barium separation in this case is 6.67 A.

With a simple model of the barium atoms forming a cubic lattice and a knowledge of the density and composition at the limit of miscibility, Levin and Block found that the average separation of barium atoms is 6.82 A. Hence, they predicted that the structure is type B.

This paper describes the determination of the Ba-Ba distance by means of X-ray diffraction radial distribution techniques. It will be shown that the structure cannot be type A and agrees with type B.

## 2. Experimental Procedures

Measurements were made with a North American Philips X-ray diffractometer, modified so that the volume defined by the cylindrical scatter shield could he evacuated. Intensities were determined at each angular position by the average of two consecutive measurements of the time taken for 6,400 counts. The maximum difference between the two measurements was 3.5 percent. Readings were taken at intervals of 0.05 in *s*=4*π* sin *θ*/*λ* in the range 0.3 to 3.5 and intervals of 0.1 in the range 3.6 to 7.9 using CuK*α* radiation with an argon filled Geiger counter, and intervals of 0.1 in the range 6.1 to 10.0 using MoK*α* radiation and a krypton filled Geiger counter.

A warmup time of 24 hr was allowed, after which the unit was kept running until all the data for each radiation were obtained. The intensity at a standard position was checked periodically to guard against fluctuations in the incident intensity.

Monochromatization of the reflected X-ray beam was accomplished by means of balanced filters [3].

**Figure 3:**
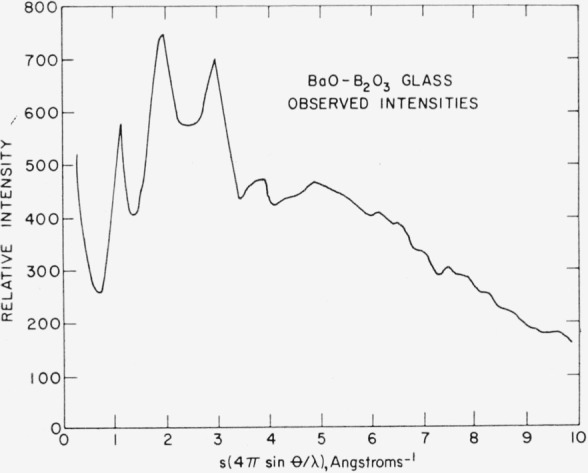
Diffraction pattern of vitreous barium borate.

The sample, supplied by G. W. Cleek and E. H. Hamilton of NBS, contained 20 mole percent BaO and 80 mole percent B_2_O_3_. The observed intensities are shown in figure 3.

## 3. Theory

Neglecting polarization and atomic constants, the amplitude of X-rays scattered from an electron distribution $\rho (\vec{R})$ is given by

$$F(\vec{k})=\int \rho (\vec{R})\;\exp(-2\pi i\vec{k}\cdot \vec{R})d\vec{R}$$ (1)

where $\vec{k}$ is the reciprocal space vector. The intensity is given by

$$I(\vec{k})=F(\vec{k})F*(\vec{k}),$$

where $F*(\vec{k})$ is the complex conjugate of $F(\vec{k})$. Then, using the convolution theorem [4], the Fourier transform of $I(\vec{k})$ is given by

$$P(\vec{R})=\int \rho (\vec{r})\rho \vec{\left(r+R\right)}d\vec{r}=\int I(\vec{k})\exp\;(2\pi i\vec{k}\cdot \vec{R})d\vec{K}$$ (2)

where, as in most X-ray diffraction transform techniques, we have assumed that the sample is of infinite extent.

If the sample is such that $I(\vec{k})$ is spherically symmetric in *k*-space, e.g., a powder or an amorphous material, the integral over the angular portions, expressing $\vec{k}$ in a spherical polar coordinate system with the *k_z_* axis parallel to $\vec{R}$, can be performed directly. In this case,

$$\begin{array}{l} P\;(\vec{R})=\int_{0}^{\infty }\int_{0}^{\pi }\int_{0}^{2\pi }I(k)\;\exp(2\pi ikR\;\cos\alpha )k^{2}\sin\alpha d\phi d\alpha \;dk\\ \;=2\pi \int_{0}^{\infty }\int_{0}^{\pi }I\;(k)\;\exp(2\pi ikR\;\cos\alpha )\;k^{2}\sin\alpha d\alpha \;dk\\ \;=(2/R)\int_{0}^{\infty }I\;(k)\;\sin(2\pi kR)\;k\;dk\\ P\;(\vec{R})=(2/4R\pi^{2})\int_{0}^{\infty }s\;I(s)\;\sin(sR)\;ds \end{array}$$ (3)

where *s*=2*πk=4π* sin *θ*λ.

Here, $P(\vec{R})$ is the three-dimensional Patterson function. Since, for a powder or an amorphous material, it is spherically symmetric, it may be written as *P*(*R*)/4*πR*^2^, where *P*(*R*) is the relative probability of finding two electrons separated by a distance *R*, and is called the electron radial distribution function. Then

$$P(R)=(2R/\pi )\int_{0}^{\infty }s\;I(s)\;\sin(sR)\;ds.$$ (4)

With the usual [5] considerations about the inability to make measurements of *I* for small *s*, and the subtraction of the contribution of each atom convoluted with itself to *P*(*R*), this can be written

$$P(R)-P_{0}(R)=(2R/\pi )\int_{s_{\min}}^{\infty }\;s\left\{I(s)-\sum_{m}{f_{m}}^{2}\right\}\;\sin(sR)\;ds$$ (5)

where $\sum_{m}f_{m}^{2}$ is the weighted sum of the squares of the atomic scattering factors of the atoms in the sample and *P*_0_(*R*) is the distribution obtained from a homogeneous array of electrons with the same average electron density as the sample.

If the usual assumption is made that the different atomic scattering factors can be expressed as multiples of a normalized average scattering factor, *f_e_*, where normalization implies *f_e_*(*s*=0)=1, then division of the integrand by the square of the normalized average scattering factor reduces the distribution to that of point atoms and yields the atomic radial distribution function commonly used [6]. This can be written

$$\sum_{m}\lambda_{m}4\pi R^{2}(\rho_{m}-\rho_{0})=(2R/\pi )\int_{s_{\min}}^{\infty }si(s)\;\sin(Rs)ds$$ (6)

where $i(s)=\left(I(s)-\sum_{m}f_{m}^{2}\right)/f_{e}^{2}$, Σ*_m_*λ*_m_* is the effective number of electrons of atom *_m_* summed over atoms *m*, and 4*πR*^2^*ρ_m_dR* is the number of atoms, each multiplied by its effective number of electrons, between *R* and *R+dR*, from atom *m*.

## 4. Calculations

The coherent scattered intensity is obtained from the experimentally measured intensity by correction for polarization and incoherent scattering. The incoherent scattering factor for barium was calculated using the equation [7]

$$I_{\mathrm{inc}}=Z-f^{2}/Z.$$ (7)

The coherent scattering factors for barium, oxygen, and boron as well as the incoherent scattering factors for boron and oxygen were obtained from standard references [8]. The incoherent scattering factors were corrected with the Breit-Dirac factor [7].

The density of the sample was 2.83 g/cm^3^ and from this value and the molecular weight, the average electron density was calculated to be 0.79 electrons/A^3^.

The data were placed on an absolute scale by setting

$$\sum_{s}sI{\left(s\right)}_{\text{obs}}\Delta s=\sum_{s}\left\{\sum_{m}s\;f_{m}^{2}+sI_{\text{calc inc}}\right\}\Delta s.$$ (8)

After corrections for polarization and incoherent scattering had been applied, the functions $s\left\{I(s)-\sum_{m}f_{m}^{2}\right\}$ and *si*(*s*) were computed from the experimental data. The integrals were replaced by sums of the form

$$\sum_{s=0.3}^{10.0}s\;i(s)\;\sin(sR)\Delta s$$

which were performed on a high speed digital computer for 0.1 A spacings in *R* from *R*=0 to *R*=9.9. Multiplication by 2*R*/*π* gave the right-hand sides of (5) and (6), and from these the radial distribution functions were calculated.

## 5. Conclusions

**Figure 4:**
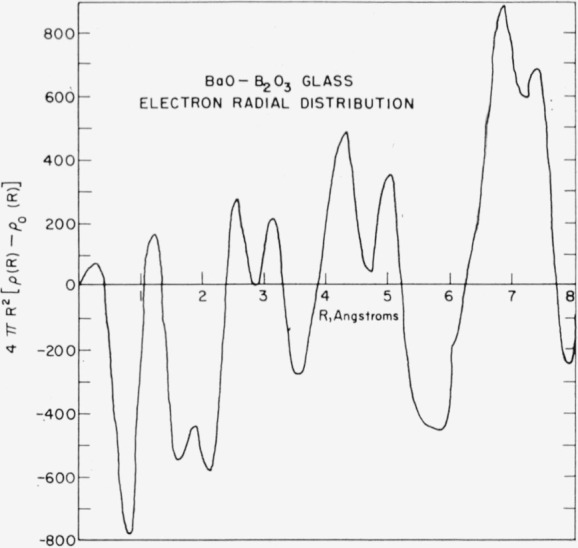
Electron radial distribution function, 4π*R*^2^[ρ(*R*)-ρ_o_(*R*)]=*P*(*R*)−*P*_0_(*R*), calculated from the data of figure 3.

Figure 4 shows the electron radial distribution *P*(*R*)−*P*_0_(*R*) obtained. The most significant features for the purpose of this study are the absence of a peak at 5.5 A and the large peak at 6.8 A. The peak at 6.8 A corresponds to the type B barium–barium separation prediction by Levin and Block. 5.5 A is the barium–barium separation for the type A structure.

**Figure 5:**
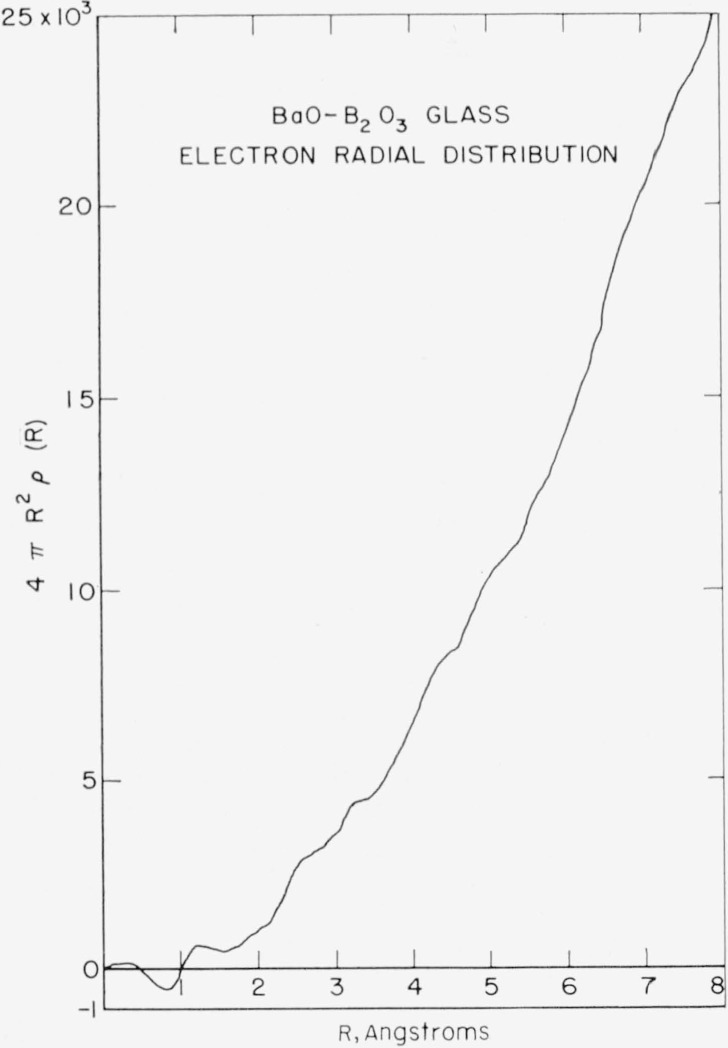
Electron radial distribution function, 4π*R*^2^ρ(*R*)=*P*(*R*), calculated from the data of figure 3.

Figure 5 shows the electron radial distribution *P*(*R*). The poor resolution does not allow for a calculation of the area under each peak. However, the areas may be calculated by determining the area between minimums of the peaks of the *P*(*R*)−*P*_0_(*R*) distribution and adding to this area the integral of *P*_0_(*R*) over the range between the minimums. This method suffers considerably in accuracy due to overlap, but offers some means of calculating areas. For this reason, the areas of the first three peaks only were calculated. The results were 322 (electrons)^2^ for the 1.2 A peak, 1750 (electrons)^2^ for the 2.55 A peak, and 2779 (electrons)^2^ for the 3.1 A peak.

**Figure 6:**
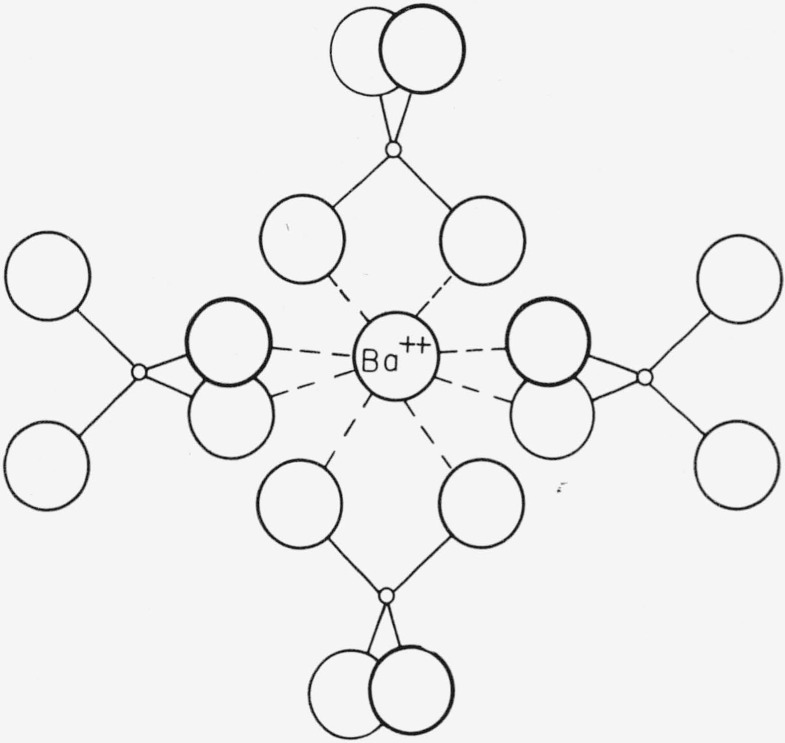
Schematic representation showing the coordination of oxygen atoms about the barium. The boron and barium configurations share edges.

A more complete picture of the type B structure is shown in figure 6. On the basis of this model, the peaks in the radial distribution can be explained readily. The 1.2 A peak corresponds to the boron–oxygen distance. The predicted area of this peak is

$$4\times 1.6\times 10\times 2\times 2=256{\left(\text{electrons}\right)}^{2}$$

Here 4 is the expected number of oxygens surrounding each boron, 1.6 is the number of borons in the basic unit used for scaling the intensities, 10 and 2 are the number of electrons per ion associated with the oxygen and boron, respectively (these values are used throughout the paper), and the last factor of 2 takes into account the fact that each peak appears twice in the distribution function. The observed area of the peak is more consistent with an oxygen coordination of 5 around boron. However, this coordination would be quite unusual and is inconsistent with the short B–O distance. Hence it seems likely that some error in the distribution function obtained from the data has changed both the area and position of the peak. In the region of small *R* this function is most susceptible to such errors and these errors will be most significant because of the small area of the peak.

The 2.55 A peak corresponds to the oxygen–oxygen separation in the boron tetrahedron. The predicted area is 1560 (electrons)^2^ which agrees to within 15 percent with the observed area of 1750 (electrons)^2^.

The 3.1 A peak appears to be a superposition of Ba–O, O–O, and Ba–B peaks. The Ba–O distance predicted on the basis of ionic radii is 2.8 A. Considering the distortion of the Ba cube, however, a larger distance is not unreasonable. The predicted area of the Ba–O peak is 1698 (electrons)^2^. This figure is based on a value of 54 scattering electrons for barium. The O–O distance predicted is 3.1 A. This distance is the longer of the edges of the distorted barium cube. The predicted area of this O–O peak is 1040 (electrons)^2^. The predicted Ba–B distance is one-half the Ba–Ba distance, or 3.4 A. The predicted area of this peak is only 173 (electrons)^2^. Thus, the peak makes no observable contribution to the radial distribution beyond its contribution to the area of the 3.1 A peak. The predicted total area of the 3.1 A peak is 2811 (electrons)^2^, in good agreement with the observed value of 2779 (electrons)^2^.

The 4.3 A peak corresponds to the distance between a barium and the closest oxygens which are not nearest neighbors. These oxygens are the parts of the boron tetrahedron which are not involved in the edge sharing with the polyhedron of the barium under consideration.

The 5.0 A peak corresponds to the distance across a face diagonal of the distorted barium cube. In addition, there should be a peak at 5.6 A corresponding to a body diagonal in the barium polyhedron. The area of this peak would be only 520 (electron)^2^. It is not observed.

**Figure 7:**
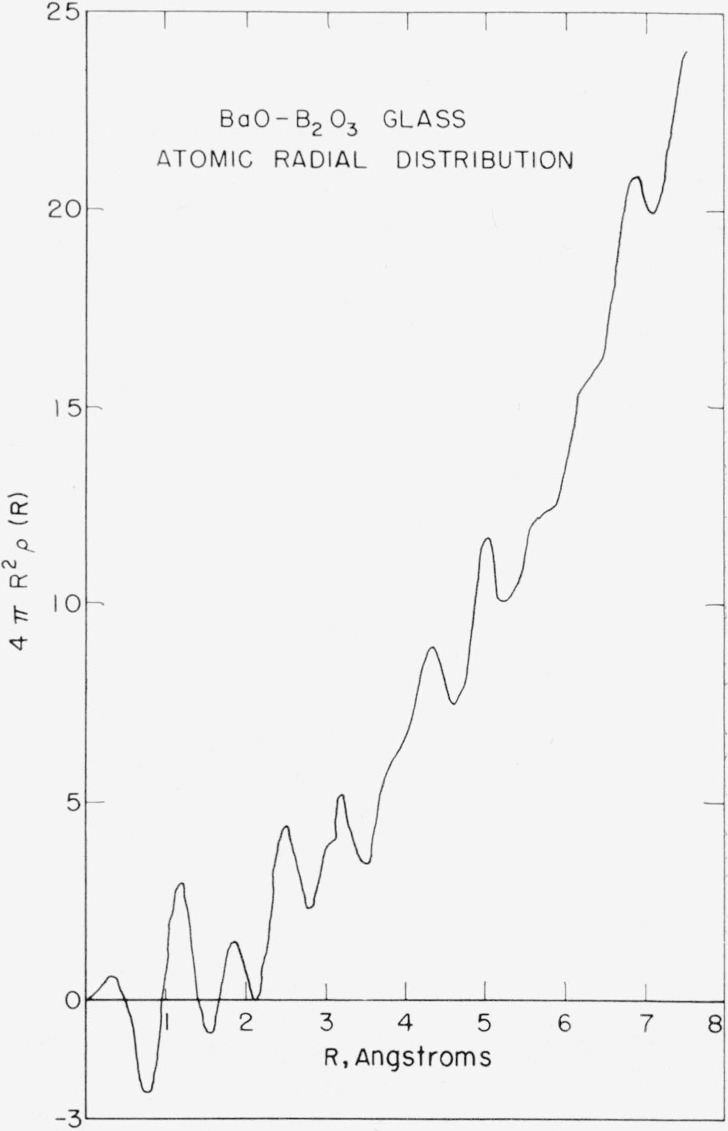
Atomic radial distribution function, 4π*R*^2^ρ(*R*), calculated from the data of figure 3.

Figure 7 shows the atomic radial distribution function obtained from the data. The most interesting feature of the peak is the clearly visible 6.8 A peak. Peaks at this distance are not usually observed in glasses. It seems reasonable to assume either that the introduction of barium into the glass has increased the range of ordering or that because of barium’s great scattering power it allowed the determination to be extended to this region.

Because of the agreement between the position of peaks on the radial distribution and the distances predicted on the basis of structure type B, it seems likely that the structure is type B and is definitely not type A. The good agreement between predicted and observed areas adds validity to this conclusion.
